# Cancer Cachexia and the Heart: A Review with Focus on China

**DOI:** 10.3390/curroncol33100635

**Published:** 2026-10-20

**Authors:** Siqi Wang, Zihao Chen, Jochen Springer

**Affiliations:** 1 Berlin Institute of Health Center for Regenerative Therapies (BCRT), Charité—Universitätsmedizin Berlin, 13353 Berlin, Germany; siqi.wang@charite.de (S.W.); zihao.chen@charite.de (Z.C.); 2 German Centre for Cardiovascular Research (DZHK) Partner Site Berlin, Charité—Universitätsmedizin Berlin, 13353 Berlin, Germany

**Keywords:** cancer cachexia, cardiac wasting, China, cardio-oncology, cardiac involvement, cachexia assessment

## Abstract

Cancer cachexia is a systemic, multifactorial syndrome characterized by progressive depletion of skeletal muscle, disruption of metabolic homeostasis, and reduced survival. In experimental models, cachexia-associated mediators, including inflammatory cytokines, myostatin-related signaling, and proteolytic activation, have been implicated in driving cardiac atrophy, interstitial fibrosis, and impaired myocardial function. Conversely, data from chronic heart failure suggest that reduced tissue perfusion, neurohormonal activation, and sustained inflammation secondary to cardiac dysfunction may further aggravate skeletal muscle wasting. These observations suggest a bidirectional relationship between cancer cachexia and cardiac dysfunction, which has not been tested in patients with cancer. Approximately 4.82 million new cancer cases occur in China annually, with a disease distribution predominated by gastrointestinal, hepatobiliary, and thoracic malignancies, all of which carry high risks of cachexia. Among 710,170 Chinese patients with cancer, 5.0% had cardiovascular disease and 13.0% had at least one cardiovascular risk factor. These figures indicate a large population at risk, but they do not show that cardiac involvement in Chinese patients differs in frequency, phenotype, or clinical impact from that in other populations; to our knowledge, no prospective data on cancer-associated cardiac involvement have been reported for Chinese patients, and cachexia assessment does not include cardiac biomarkers or echocardiographic measures. This review summarizes cancer cachexia epidemiology in China, shows the evidence linking cachexia to cardiac wasting, and evaluates how cardiac dysfunction may in turn influence the cachectic trajectory. Accordingly, we outline the key clinical and mechanistic questions that remain unanswered in Chinese patients and propose a research agenda in which cardiac assessment is evaluated prospectively before it is considered for routine care.

## Simple Summary

Depending on the type of cancer, up to 80% of cancer patients develop cachexia, a progressive loss of fat and muscle mass. Cachexia has profound negative effects on morbidity and mortality with cachexia being the cause of death in approx. 20% of cancer-related deaths. The loss of muscle mass is not restricted to skeletal muscle alone, but also strongly affects the heart, with loss of tissue having been reported to be greater in the heart compared to skeletal muscle in experimental cancer cachexia models. In patients with advanced cancer, reduced left ventricular mass has been associated with lower stroke volume, although left ventricular ejection fraction, the standard measure of cardiac function, is frequently preserved. Routine cardiac assessment may therefore fail to detect this loss of left ventricular mass, whose effect on organ perfusion is unknown. This review summarizes the evidence, focusing on China, and identifies questions for future research.

## 1. Introduction

Cancer cachexia is characterized by skeletal muscle wasting and adipose tissue loss, but its pathological consequences are not only confined to these tissues. Experimental data indicates that inflammatory, proteolytic, and anti-anabolic signals implicated in skeletal muscle atrophy similarly underlie cardiac atrophy, fibrosis, and functional impairment, albeit through different proteolytic mechanisms [1,2,3]. Compromised cardiac function may in turn intensify fatigue, worsen exercise intolerance, and reduce tissue perfusion, adding to the clinical burden of cachexia. These findings together support a broader, multiorgan conceptualization of cancer cachexia.

National epidemiological data from China indicate approximately 4.82 million incident cancer cases and 2.57 million cancer-related deaths in 2022 [4], with gastrointestinal, hepatobiliary, and thoracic malignancies being the predominant tumor types, all of which are strongly associated with cachexia [4,5]. China therefore represents one of the largest populations globally at risk for cancer cachexia. Considering this burden, Chinese investigators have actively advanced the epidemiological and diagnostic understanding of cancer cachexia spanning national prevalence estimates [6], validation of Asian-adapted diagnostic criteria [7,8], and machine-learning approaches for case identification in real-world settings [9]. Despite these advances, cancer cachexia and cardio-oncology have been studied as separate fields in China. Cachexia research has largely focused on nutritional epidemiology and diagnostics, whereas cardio-oncology literature has mainly addressed treatment-related cardiotoxicity and risk surveillance. The potential interplay between systemic cachexia and cardiac dysfunction has not been characterized in Chinese cohorts. This review integrates evidence on cancer cachexia with emerging data on cardiac involvement to delineate the key clinical and mechanistic questions that remain unaddressed in this field. Particular attention is paid to China in view of the large number of patients at risk and the lack of reported cardiac data from Chinese cachexia cohorts; however, the available data do not indicate that cardiac involvement in Chinese patients differs in phenotype or clinical impact from that in other populations. Throughout the review, the evidence is classified as human clinical data, preclinical data, or mechanistic hypotheses.

This narrative review is not based on a systematic literature search. The cited studies were selected for their relevance to cardiac involvement in cancer cachexia, with emphasis on clinical studies including cardiac endpoints, studies of cachexia prevalence or diagnosis in China, and experimental studies on cardiac wasting. As this selection may be incomplete, statements that data have not been reported refer only to the studies identified by the authors.

## 2. Cancer Cachexia in China: Epidemiology, Cardiovascular Comorbidity, and the Diagnostic Gap

### 2.1. Cachexia-Prone Cancers and Coexisting Cardiovascular Burden in China

China’s cancer burden is dominated by malignancies with high risk of cachexia, particularly lung, gastric, liver, esophageal, and colorectal cancers, which act as the leading contributors to cancer incidence and mortality [4]. Cachexia prevalence varies by tumor site, being highest in cancers of the pancreas, esophagus and stomach, intermediate in lung cancers, and relatively low in breast and prostate cancers [10]. In these high-prevalence cancers, reduced food intake arises from local effects of the tumor on the alimentary tract, such as dysphagia, gastrointestinal obstruction, and early satiety; from anorexia driven by tumor-induced inflammation and the gastrointestinal toxicity of anticancer treatment [10]; and from treatment-induced alterations in taste perception that further blunt appetite [11]. These mechanisms are not specific to China; their relevance to China lies in the large absolute number of patients with these tumor types.

Further, this vulnerability is compounded by the burden of cardiovascular comorbidity. Across a large multicenter Chinese cohort of 710,170 patients with cancer, 13.0% of them had at least one cardiovascular risk factor and 5.0% had cardiovascular disease; all-cause mortality was higher in both groups than in patients without these conditions, and in multivariable analysis most individual conditions, except dyslipidemia, were associated with higher mortality [12]. In another separate single-center cohort of 35,861 Chinese patients with major solid tumors, hypertension was present in 21.9% and coronary heart disease in 6.5% [13]. These data indicate that cardiovascular comorbidity is common among Chinese patients with cancer. They do not, however, show whether cachexia-related cardiac changes in Chinese patients differ in frequency, phenotype, or prognostic impact from those reported in other populations; we are not aware of studies that have investigated this question.

### 2.2. Cachexia Prevalence and the Absence of Cardiac Assessment

Cancer cachexia is highly prevalent in Chinese patients with advanced cancer. A single-centre survey of 390 patients with advanced cancer, published in 2015, reported a prevalence of 35.9%, highest in pancreatic (88.9%), gastric (76.5%), and esophageal cancers (52.9%); 76.4% of cachectic cases were not recognized by the treating physicians [14]. A subsequent national estimate placed the prevalence at 37.0% [6], and a post hoc analysis of the INSCOC cohort (*n* = 5426) using AWGC2023 criteria reported 43.8% [7]. At this scale of disease burden, even if cardiac wasting affects only a proportion of cachectic patients, the absolute number is substantial.

The diagnosis of cachexia in China has been advanced through validation of the AWGC framework [7,8] and machine-learning tools for cases with incomplete weight-loss data [9,15]. These advances, however, are still narrowly focused on recognizing manifestations of cachexia alone; cardiac biomarkers, echocardiography, and functional cardiac assessment are not taken into consideration, although data from other countries indicate that cardiac involvement in patients with advanced cancer is associated with patient outcomes and is frequently undetected. In a prospective German study of 300 patients with advanced cancer, low left ventricular mass was independently associated with all-cause mortality and was not explained by prior cardiotoxic therapy [16]. Separately, among 70 treatment-naive patients with lung cancer in Canada, 12.9% had previously unrecognized cardiac disorders and a further 14.3% had diastolic dysfunction with preserved ejection fraction, both associated with aggravated fatigue and dyspnea [17]. To our knowledge, comparable data have not been reported for Chinese patients.

## 3. Cardiac Wasting as a Component of a Multiorgan Syndrome

Cancer cachexia is now regarded not only as a disorder of skeletal muscle and adipose tissue, but as a multiorgan syndrome driven by systemic cachexia-associated signals. Mechanistically, its pathogenesis encompasses chronic inflammation, tumor-derived humoral factors, neurohormonal dysregulation, altered hepatic metabolism, intestinal barrier dysfunction, adipose tissue remodeling, and centrally mediated disturbances of appetite and energy homeostasis [5,18]. Furthermore, Wang et al. have described cancer cachexia as a disorder of inter-organ communication, emphasizing that cachexia-related factors disrupt signaling across organ axes including the brain–liver–gut–muscle and brain–pituitary–adrenal–muscle pathways [18]. Collectively, these organ-specific alterations are considered to reflect a shared pathological process rather than independent processes [19].

Cardiac alterations in cancer cachexia have been less extensively investigated. Whereas the involvement of skeletal muscle, liver, gut, and the hypothalamus in cachexia has been characterized largely in terms of altered nutritional and metabolic homeostasis [20,21], cardiac wasting in patients with advanced cancer has been associated with structural and hemodynamic alterations, including lower stroke volume and cardiac output, as well as with impaired functional capacity and increased mortality [16,22]; whether cardiac wasting progresses to a heart failure–like syndrome remains to be determined. In 2023, a prospective German cohort of 300 patients with advanced cancer demonstrated that LV mass was reduced relative to healthy controls, with the greatest reduction observed in cachectic patients; low LV mass was associated with poor functional status and was an independent predictor of all-cause mortality [16]. In the subset of patients who underwent repeat echocardiography (*n* = 90; mean interval ≈ 4 months), LV mass declined by a further 9.3% ± 1.4%, and those developing cardiac wasting showed a significant fall in stroke volume alongside a compensatory rise in resting heart rate, consistent with progressive impairment of cardiac performance [16]. A subsequent echocardiographic phenotyping study, stratifying advanced-cancer patients by LV mass, characterized low LV mass as a cardiomyopathy in which smaller chamber volumes, ventricular wall thinning, lower stroke volume, and reduced cardiac output occur despite largely preserved LVEF and global longitudinal strain [22]. Together, these findings indicate that LVEF and GLS may fail to capture the full extent of cardiac impairment in patients with advanced cancer.

The association of low LV mass with all-cause mortality [16] suggests that cardiac wasting has prognostic significance, although it was observed in a single-centre cohort. In experimental models, cancer cachexia has been shown to induce cardiac mass loss with upregulation of the ubiquitin–proteasome system [1,23], and this cardiac involvement is further accompanied by intrinsic cardiomyocyte contractility and calcium-handling deficits [24], with myocardial fibrosis likewise reported in preclinical models [2,24,25], which has been attributed to inflammatory cytokines as well as to dysregulated anabolic and catabolic signaling. In a rat hepatoma model, bisoprolol and spironolactone reduced LV mass loss, attenuated cardiac dysfunction, and improved survival [25], although the translational relevance of these findings has not been established in clinical trials. On the basis of these clinical associations and experimental findings, cardiac wasting may represent a distinct manifestation of cancer cachexia, although this remains to be confirmed in larger clinical cohorts; a multi-biomarker approach to its detection has been proposed, and dedicated attention in future clinical trials has been called for [26].

At present, there is no consensus definition of cardiac wasting in patients with cancer. In the clinical studies discussed above, cardiac wasting was defined operationally by echocardiographic LV mass: cross-sectionally as LV mass, or LV mass indexed to height squared, below sex-specific cut-offs [16,22], and longitudinally as a decrease in LV mass of 10% or more over 3 to 12 months, which was associated with impaired functional status and reduced survival [16,22]. For this review, the term cardiac wasting refers to low or declining LV mass in patients with cancer according to these criteria [16,22], whereas the term cardiac dysfunction refers to impaired cardiac structure or function of any cause. This definition is applied throughout the review. The term cardiac atrophy is used for a reduction in heart weight, LV mass, or cardiomyocyte size in experimental models, and the term cardiac involvement refers to cardiac wasting or cardiac dysfunction in patients with cancer cachexia. Cardiac wasting defined in this way differs from cancer therapy-related cardiac dysfunction (CTRCD). According to the 2022 European Society of Cardiology (ESC) guidelines on cardio-oncology, CTRCD is defined by symptomatic heart failure or, in asymptomatic patients, by a new decline in LVEF to below 50%, a new relative decline in GLS of more than 15% from baseline, and/or a new rise in cardiac troponin or natriuretic peptides in association with anticancer therapy [27]. Because LVEF and GLS were largely preserved in patients with low LV mass [22], cardiac wasting may not fulfil the criteria for CTRCD, and the presence of CTRCD does not in itself indicate cardiac wasting.

Other causes of cardiac dysfunction must also be considered in patients with cancer. These include (i) pre-existing cardiovascular disease, such as coronary artery disease, valvular heart disease, and heart failure; (ii) cancer-related cardiac and hemodynamic abnormalities unlikely to be caused by wasting, such as those detected in treatment-naive patients with non-small cell lung cancer, including reduced LVEF (likely due to pre-existing ischemic heart disease in seven patients), severe right ventricular dilation with pulmonary hypertension, and pericardial effusion requiring drainage [17]; (iii) CTRCD, as described above [27]; (iv) anemia, which is common in patients with cancer and impairs myocardial oxygen balance; in mice with advanced ovarian cancer, anemia was accompanied by lower heart rate and cardiac output [28]; and (v) non-cardiac systemic conditions, such as infection, which was an exclusion criterion in the clinical studies of cardiac wasting [16,22], and severe malnutrition, which has been associated with reduced myocardial mass [29]. Systemic inflammation frequently accompanies cachexia [5], so that its effects on the heart cannot readily be separated from those of cachexia (Section 4.1). In addition, chronic heart failure can itself lead to cachexia [30,31]; the temporal sequence of weight loss and cardiac change therefore needs to be determined before cardiac changes are attributed to cancer cachexia. Both clinical studies that characterized cardiac wasting in cancer were conducted at the same centre with overlapping recruitment periods, and both excluded patients with significant cardiovascular disease, an LVEF below 50%, or active infection [16,22]. The current operational definition is therefore derived from a selected population without these conditions, and its applicability to patients with cardiovascular comorbidity, which is common among Chinese patients with cancer [12,13], remains unclear. In the German cohort, the association between low LV mass and all-cause mortality persisted after adjustment for cardiotoxic anticancer therapy, hemoglobin, C-reactive protein, NT-proBNP, and high-sensitivity troponin T [16]. Future studies should nevertheless record these conditions, including hemoglobin concentration, markers of inflammation, and exposure to cardiotoxic treatment, so that their contribution can be considered.

In China, the scale of the cancer burden, the predominance of tumor types strongly associated with cachexia, and the high prevalence of cardiovascular comorbidity (Section 2) indicate that a large number of Chinese patients may be at risk of cardiac wasting. These population-level data, however, do not indicate whether its frequency, phenotype, or clinical impact in Chinese patients differ from those reported in other populations. Whether cachexia exerts direct cardiac effects, compromising cardiac structure or function independent of this baseline cardiovascular burden, remains poorly studied. Whereas existing evidence on cancer-related cardiac wasting comes almost entirely from Western cohorts [16,26], data from Chinese patients are scarce; thus, the frequency of cardiac involvement in patients with cancer cachexia in China, whether it can be identified at a subclinical stage, and whether its recognition could refine risk stratification or inform clinical management remain unanswered.

## 4. Molecular and Cellular Mechanisms of Cancer Cachexia–Associated Cardiac Wasting

### 4.1. Proteolytic Pathways and Their Inflammatory Regulation in Cachexia-Associated Cardiac Wasting

Unless otherwise stated, the data summarized in this section were obtained in rodent models or in cell culture, and human data are identified as such. Table 1 summarizes the type of evidence for the principal findings. Delineating the molecular basis of cachexia-induced cardiomyocyte atrophy and cardiac dysfunction is necessary to identify early detection strategies and therapeutic targets in this population. The ubiquitin–proteasome system and the autophagy–lysosomal pathway, both of which promote skeletal muscle wasting in cancer cachexia [1], are also activated in cardiac muscle in rodent models of cancer cachexia. Single-nucleus transcriptomic profiling of atrophying limb muscle in tumor-bearing mice has shown upregulation of the atrogenes atrogin-1 and MuRF-1 within myonuclei, together with activation of NF-κB and JAK–STAT signaling [32]. Using the C26 adenocarcinoma model, Wysong et al. demonstrated that cardiac MuRF-1 and atrogin-1 expression peaked at approximately day 10 after tumor implantation and returned to baseline by day 17 [33]. In a C26 tumor-bearing mouse model, Tian et al. found increased cardiac MuRF-1 (mRNA and protein) and atrogin-1 (mRNA only; atrogin-1 protein was not assessed), accompanied by a 43% reduction in myosin heavy chain, a 58% reduction in troponin I, and increased protein ubiquitination, consistent with ubiquitin–proteasome-mediated cardiac atrophy [23]. In other rodent models of cancer cachexia, cachectic hearts showed increased expression of Beclin-1 and LC3, consistent with upregulation of autophagy-related signaling [1].

Although both pathways are engaged in skeletal and cardiac muscle, their upstream regulation appears to be tissue specific. In skeletal muscle, impaired Akt signaling activates FoxO1, which induces the E3 ubiquitin ligase atrogin-1 and thereby drives proteasomal degradation [34]. In the heart, by contrast, FoxO3 has been reported to promote atrophy mainly through the autophagy–lysosomal pathway rather than the proteasome, although this was shown in a non-cancer model [1]. It has been proposed that the high basal proteasomal activity of the heart, which reflects its higher metabolic rate and protein turnover at rest compared with skeletal muscle, leaves limited capacity for further upregulation, so that induction of the autophagy–lysosomal pathway may be sufficient to mediate myocardial protein degradation [1]. Taken together, these data from rodent models indicate that cancer cachexia activates both the ubiquitin–proteasome and autophagy–lysosomal pathways in the heart, whereas the predominant pathway and its upstream regulators appear to differ from those operating in skeletal muscle; neither observation has been confirmed in human myocardium.

In the C26 model, inflammatory mediators released by tumor and immune cells, including TNF-α and IL-6, have been proposed to activate NF-κB signaling in cardiac muscle; whether NF-κB activation directly drives the transient induction of MuRF-1 and atrogin-1 in the heart remains to be confirmed [33]. In skeletal muscle, IL-1 has been shown to induce atrogin-1/MAFbx and MuRF-1 mRNA expression in cultured myotubes [35]. MuRF-1 is transcriptionally regulated by IKKβ/NF-κB signaling, whereas atrogin-1/MAFbx appears largely NF-κB-independent [36]. FoxO3 can induce both ligases [37]. Inflammatory and catabolic signaling pathways have thus been implicated in the regulation of atrogin-1/MAFbx and MuRF-1 in skeletal muscle [38], although it is unclear whether similar mechanisms operate in cardiac tissue. In a mouse model of pancreatic cancer, leukocyte infiltration of the diaphragm preceded muscle wasting and fibrotic remodeling, indicating that local inflammation can precede overt muscle atrophy [39]. Using the same C26 model, Wysong et al. showed that NF-κB blockade with Compound A or the NEMO-binding domain peptide prevented the 24.3% reduction in LV mass (normalized to tibia length) otherwise seen in tumor-bearing mice at day 17, and improved fractional shortening and ejection fraction towards control values, supporting a role for NF-κB in cachexia-induced cardiac atrophy [33].

Whether the inflammatory pathways implicated in rodent models of cachexia-induced cardiac atrophy operate similarly in patients remains unknown. Clinical data show higher circulating IL-6 levels in cachectic than in non-cachectic cancer patients, and among patients with follow-up echocardiography, baseline IL-6 was significantly higher in those who subsequently developed an LV mass reduction ≥ 10% than in those with a reduction < 5% [16]. These findings support an association between systemic inflammation and cardiac wasting but are insufficient to establish causality; it remains unclear whether IL-6 directly contributes to LV mass loss or instead reflects tumor- or host-related inflammatory activity [16].

### 4.2. Impaired Anabolic Signaling, Metabolic Remodeling, and Mitochondrial Dysfunction in Cachexia-Associated Cardiac Wasting

In addition to proteolytic pathway activation, suppression of anabolic signaling may also contribute to loss of LV mass in cancer cachexia. In ApcMin/+ mice, cardiac wasting is associated with reduced myofibrillar protein synthesis and decreased phosphorylation of mTOR and its downstream targets, including S6 ribosomal protein and 4EBP1, despite paradoxically increased Akt phosphorylation, indicating that mTOR suppression in this model occurs independently of Akt [40]. In AH-130 tumor-bearing rats, cardiac IGF-1R expression is markedly reduced, accompanied by decreased phosphorylation of Akt and GSK-3β [25]. The mechanistic basis of mTOR suppression thus differs between models, with upstream anabolic signaling intact in the ApcMin/+ heart but impaired at the level of IGF-1R and Akt in the AH-130 model.

In skeletal muscle, the myostatin/activin A–ACVR2B axis suppresses anabolic signaling through inhibition of Akt/mTORC1 and promotes FoxO1-dependent UPS activation [34]. Whether this pathway operates similarly in the heart is uncertain: in AH-130 tumor-bearing rats, myocardial myostatin expression was elevated 3.1-fold compared with sham animals, yet FoxO and MuRF-1 were not correspondingly upregulated [25]. In a separate rat model of mammary tumorigenesis, cardiac phosphorylated Smad3 was likewise increased, consistent with activation of myostatin canonical signaling in the heart; however, a comparable increase occurred in exercised tumor-free controls, indicating this change is not specific to tumor burden [41]. In human cancer patients, circulating activin A is elevated in cachexia, whereas myostatin is, in contrast, decreased [42]. Since these measurements reflect circulating levels and skeletal muscle rather than the myocardium, whether myostatin contributes to cardiac wasting in cancer patients warrants further investigation.

Beyond proteolytic and anabolic signaling dysregulation, metabolic remodeling has also been linked to cardiac dysfunction in rodent models of cancer cachexia. In C26 tumor-bearing mice, cardiac PPARα and CPT1β mRNA expression were significantly reduced alongside a shift from MHCα to MHCβ and re-expression of fetal markers including BNP, indicating reduced fatty acid oxidative gene expression and pathological cardiac remodeling [23]. Reduced levels of the contractile proteins MHC and troponin I were observed in the same model, associated with elevated MuRF-1 and atrogin-1 expression and increased protein ubiquitination, suggesting ubiquitin–proteasome-mediated degradation potentially regulated via p44/42 MAPK signaling [23]. It has been suggested that elevated IL-6 may impair cardiac function by reducing fatty acid oxidation and mitochondrial function [29]; whether this adds to the contractile deficit caused by sarcomeric protein loss has not been tested. Tumor-derived secreted factors, termed cachexokines, have been shown to trigger atrophy and aberrant fatty acid metabolism in cultured cardiomyocytes. Among a panel of candidate cachexokines derived from colon cancer cells, engineered secretion of Ataxin-10 from non-cachexia-inducing cells was sufficient to induce cachexia phenotypes in cardiomyocytes, and circulating Ataxin-10 was elevated in mice bearing cachexia-inducing tumors and in patients with cancer cachexia [43].

In rodent models, the fetal gene program described above is accompanied by mitochondrial dysfunction in the cachectic heart. The shift from MHCα to MHCβ and the concurrent GLUT4-to-GLUT1 switch reflect a departure from fatty acid-supported oxidative phosphorylation towards increased glycolytic dependence in cardiomyocytes; this isoform transition is associated with systolic dysfunction and overall deterioration of cardiac performance [23,44]. In C26 tumor-bearing mice, cardiac mitochondria show increased reactive oxygen species production and mitochondrial uncoupling; treatment with the mitochondria-targeting peptide SS-31, which stabilizes cardiolipin and reduces oxidative damage to the inner mitochondrial membrane, significantly attenuated left ventricular dysfunction in tumor-bearing animals, and prevention of mitochondrial dysfunction alone was sufficient to preserve cardiac function in this model [45]. In an orthotopic model of ovarian cancer, fatty acid-supported mitochondrial respiration was reduced in left ventricular tissue of tumor-bearing mice while carbohydrate-supported respiration increased, a substrate shift also seen in heart failure; the patient-derived pancreatic xenograft model in the same study instead showed myosin heavy chain isoform switching and upregulation of autophagy/ubiquitin–proteasome markers [28]. A proposed reduction in myocardial oxygen delivery, secondary to capillary rarefaction and/or anemia, may underlie this substrate shift and further limit ATP supply in the cachectic heart, exacerbating the contractile deficit arising from sarcomeric protein degradation. This mechanism was proposed on the basis of anemia and a non-significant decrease in myocardial capillary density (*p* = 0.07) in mice with orthotopic ovarian cancer and has not been tested directly [28]. 

### 4.3. Tumor-Derived Mediators and Structural and Functional Cardiac Changes

Beyond proteolytic and metabolic remodeling, cardiomyocyte function in experimental cachexia can also be impaired through additional mechanisms; the relative contribution of cell-intrinsic defects versus specific circulating mediators is unclear: in a C26 adenocarcinoma mouse model, cardiomyocytes from cachectic animals exhibited contractility deficits attributable in part to impaired intracellular calcium cycling, persisting even when myocytes were isolated from the catabolic and inflammatory environment of the whole animal; the upstream molecular basis of the calcium-handling defect itself remains to be defined [24]. In contrast, a specific tumor-derived mediator has been identified in a colorectal cancer model: HMGB1, secreted by tumor cells, binds RAGE and TLR4 on cardiomyocytes, activating NF-κB while downregulating PI3K and phosphorylated AKT expression, thereby relieving the autophagy-inhibitory brake and promoting apoptosis and autophagy; neutralizing HMGB1 in cardiomyocytes exposed to ascites from a rat cachexia model mitigated this damage [46]. RAGE, a receptor for HMGB1 and other ligands, is absent from healthy adult myofibers but is re-expressed in atrophying myofibers under cancer conditions; myofiber-specific RAGE ablation attenuates muscle wasting and inflammation in tumor-bearing mice, indicating that myofiber-level RAGE signaling contributes to cancer-associated muscle loss [47].

In a C26 adenocarcinoma mouse model, tumor-bearing mice show reduced fractional shortening, increased myocardial fibrosis, disrupted sarcomeric and mitochondrial ultrastructure, and reactivation of the fetal myosin heavy chain gene program [48]. In experimental models, early LV atrophy is similarly accompanied by wall thinning and chamber dilation, and systolic and diastolic dysfunction emerge in later stages, followed by increased fibrosis [2]. In patients with advanced cancer, low LV mass with a further decline during follow-up has been reported [16]; previously undiagnosed cardiac abnormalities have also been identified in treatment-naive patients with lung cancer [17] (Section 2.2).

**Figure 1:**
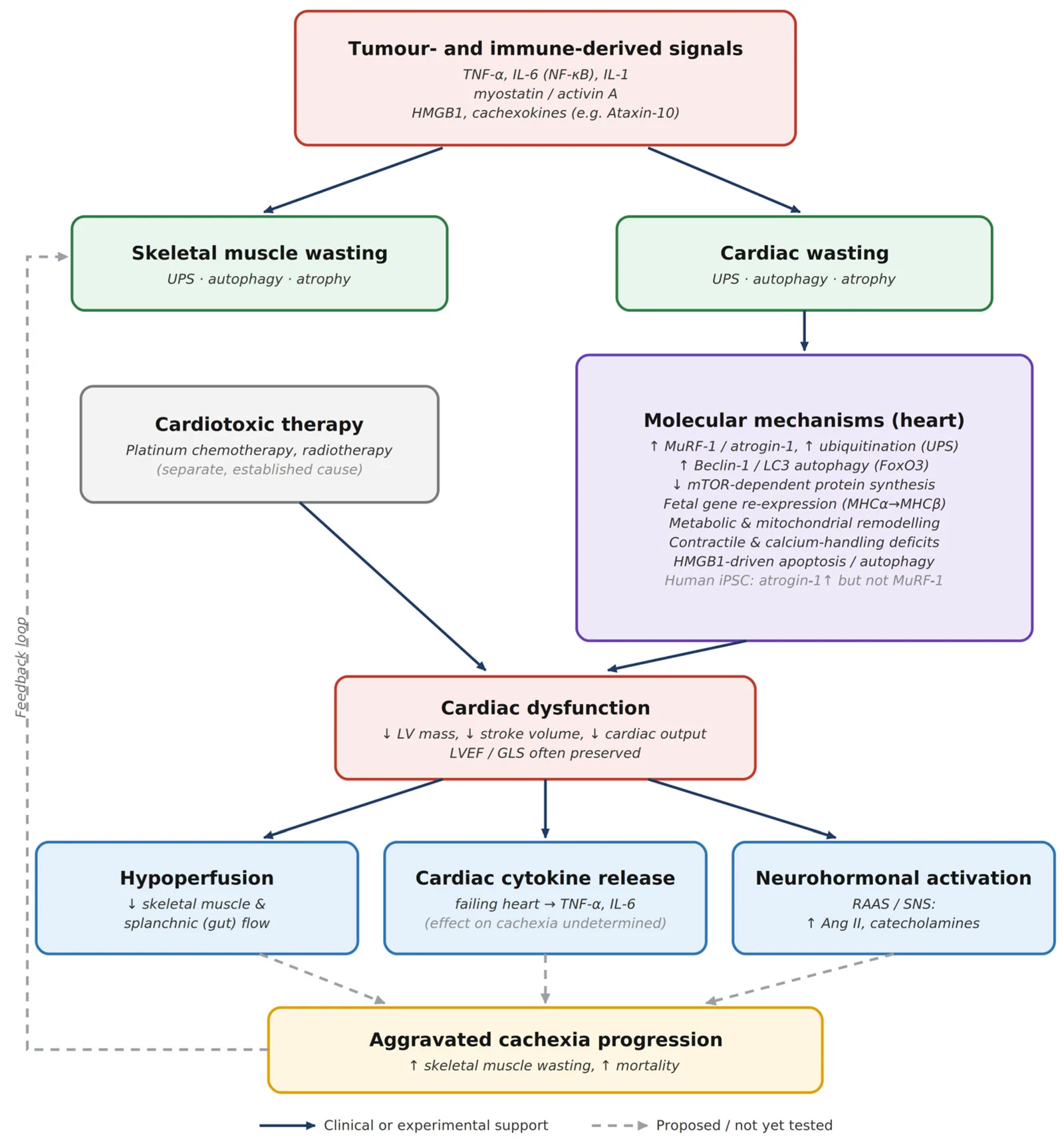
Mechanisms underlying cardiac wasting and dysfunction in cancer cachexia. Tumor- and immune-derived mediators (inflammatory cytokines such as TNF-α, IL-6, and IL-1, the myostatin/activin A axis, HMGB1, and cachexokines) drive wasting in skeletal muscle and, in the heart, contribute to both wasting and dysfunction. Within the heart, largely rodent evidence implicates several converging processes: activation of the ubiquitin–proteasome and autophagy pathways, reduced mTOR-dependent protein synthesis, metabolic and mitochondrial remodeling, contractile and calcium-handling deficits, as well as HMGB1-induced injury. Collectively, these processes lower left ventricular mass, stroke volume, and cardiac output, whereas LVEF and global longitudinal strain are often preserved. Separately, platinum-based chemotherapy and radiotherapy also contribute to cardiac dysfunction. Furthermore, cardiac dysfunction may, in turn, aggravate cachexia through hypoperfusion, cardiac cytokine release, and RAAS/sympathetic activation; these feedback links (dashed) are proposed but not yet tested in patients with cancer cachexia. Solid arrows indicate pathways with clinical or experimental support; dashed arrows indicate proposed mechanisms. Box colors indicate the level of the pathway: red, tumor- and immune-derived signals or cardiac dysfunction; green, tissue wasting; purple, molecular mechanisms in the heart; grey, cardiotoxic therapy as a separate cause; blue, proposed consequences of cardiac dysfunction; orange, aggravated cachexia progression. Text in grey denotes qualifying notes. The molecular mechanisms shown are derived mainly from rodent and cell culture models; human evidence consists mainly of observational associations of LV mass, stroke volume, and circulating IL-6 with functional status and survival [16,22] (Table 1). In human iPSC-derived cardiac muscle, atrogin-1, but not MuRF-1, was upregulated after cachexia induction [55].This schematic was drafted with the assistance of a generative AI tool (Claude Fable 5, Anthropic) and was subsequently reviewed, corrected and edited by the authors, who take full responsibility for its content; the prompt used is provided in the Supplementary Materials (Text S1). ↑, increased; ↓, decreased.

Taken together, preclinical studies have proposed several distinct mechanisms through which cancer cachexia may lead to cardiac atrophy and dysfunction (Figure 1): elevated MuRF-1 and atrogin-1 expression, increased protein ubiquitination, and decreased sarcomeric protein content, indicating that ubiquitin–proteasome-mediated proteolysis may in part contribute to sarcomeric protein loss [23]; Beclin1 upregulation (cardiac-specific) alongside LC3 induction, occurring independently of ubiquitination or apoptotic activation, with FoxO3 reported, in a non-cancer model, to drive cardiac atrophy via the autophagy-lysosomal pathway in contrast to its proteasome-dependent role in skeletal muscle [1]; suppression of Akt/mTOR-dependent protein synthesis [25]; cardiac remodeling including fetal gene re-expression (MHCα to MHCβ isoform switch at the protein level) [23,48]; metabolic reprogramming with reduced expression of PPARα and its target gene CPT1β [23], although this PPARα downregulation appears model-dependent and was not reproduced at the protein level in an LLC model [49]; cell-intrinsic contractile and calcium-handling deficits independent of systemic factors [24]; and direct induction of apoptosis and autophagy in cardiomyoblasts by the tumor-derived factor HMGB1 [46]. Whether these mechanisms operate similarly in humans is unknown; available clinical data from a follow-up subset support an association between elevated baseline IL-6 and subsequent LV mass decline, but do not demonstrate causality [16].

**Table 1:** Type of evidence for the principal findings discussed in Section 3, Section 4 and Section 5.

Finding	Type of Evidence	Model or Population	Ref.
*Human clinical data in patients with cancer*			
Lower LV mass than in healthy controls, most pronounced in cachexia; low LV mass associated with poor functional status and independently with all-cause mortality	Prospective observational cohort	300 patients with advanced cancer (Germany)	[16]
Further decrease in LV mass (9.3% ± 1.4%) over approximately 4 months; lower stroke volume and higher resting heart rate in patients developing cardiac wasting	Prospective cohort, follow-up subset	90 patients with repeat echocardiography	[16]
Low LV mass phenotype: smaller chamber volumes, thinner walls, lower stroke volume and cardiac output, largely preserved LVEF and GLS	Echocardiographic phenotyping study	Patients with advanced cancer grouped by LV mass quartiles	[22]
Cardiac abnormalities in 27.1% of patients: clinically relevant cardiac disorders in 12.9% (previously unknown in 8 of 9) and diastolic dysfunction with preserved LVEF in 14.3%; associated with fatigue and dyspnea	Cross-sectional study	70 treatment-naive patients with metastatic non-small cell lung cancer (Canada)	[17]
Higher baseline IL-6 in patients with subsequent LV mass loss ≥ 10% than in those with loss < 5%	Observational association; causality not established	Follow-up subset of the cohort in [16]	[16]
Higher circulating activin A and lower circulating myostatin in cachexia	Circulating biomarkers; no myocardial data	Patients with cancer	[42]
Human data from non-cancer settings (applied to cancer cachexia by analogy)			
Reduced leg blood flow in parallel with reduced cardiac output; RAAS and sympathetic activation; myocardial TNF-α expression; circulating IL-6 as a predictor of mortality	Clinical and tissue studies in heart failure	Patients with chronic heart failure, including cardiac cachexia	[30,31,50,51,52,53,54]
Human cell model			
Upregulation of atrogin-1 but not MuRF-1 after cachexia induction	Human iPSC-derived cardiac muscle; preprint, not peer reviewed	C26- and HCT116-induced cachexia models	[55]
Preclinical in vivo data			
Ubiquitin–proteasome activation (↑MuRF-1, ↑atrogin-1, ↑ubiquitination) with loss of myosin heavy chain and troponin I	Animal, in vivo	C26 tumor-bearing mice	[23,33]
Autophagy–lysosomal activation (↑Beclin-1, ↑LC3) without significant induction of ubiquitination or apoptosis; FoxO3-induced cardiac atrophy via this pathway reported in a non-cancer model	Animal, in vivo (review)	Rodent cachexia models; FoxO3 data from a non-cancer model	[1]
Prevention of LV mass loss and dysfunction by NF-κB blockade	Animal, intervention	C26 tumor-bearing mice	[33]
Reduced mTOR-dependent protein synthesis; reduced IGF-1R and Akt phosphorylation	Animal, in vivo	ApcMin/+ mice; AH-130 tumor-bearing rats	[25,40]
Increased myocardial myostatin expression or Smad3 phosphorylation	Animal, in vivo	AH-130 tumor-bearing rats; rat mammary tumor model	[25,41]
Metabolic and mitochondrial remodeling (↓PPARα and CPT1β, MHC isoform switch, increased mitochondrial reactive oxygen species, impaired mitophagy, shift from fatty acid- to carbohydrate-supported respiration)	Animal, in vivo	C26, Lewis lung carcinoma, orthotopic ovarian cancer, and pancreatic xenograft models	[23,28,45,49]
Engineered Ataxin-10 secretion sufficient to induce cachexia phenotypes in cardiomyocytes; circulating Ataxin-10 elevated in tumor-bearing mice and in patients with cancer cachexia	In vitro; circulating levels in mice and patients	Colon cancer cell-derived factors; cardiomyocytes	[43]
Myocardial fibrosis, wall thinning, and systolic and diastolic dysfunction	Animal, in vivo	C26 tumor-bearing mice; AH-130 tumor-bearing rats; rodent models (review)	[2,24,25,48]
Attenuation of cardiac wasting or dysfunction by bisoprolol, spironolactone, SS-31, or soluble ActRIIB-Fc	Animal, intervention; not tested in clinical trials	AH-130 tumor-bearing rats; C26 tumor-bearing mice	[25,45,56]
Ex vivo and in vitro data			
Contraction and relaxation deficits in ex vivo perfused hearts; contractility and calcium-handling deficits in isolated cardiomyocytes	Ex vivo	Hearts and cardiomyocytes from C26 tumor-bearing mice	[24]
HMGB1-induced apoptosis and autophagy via RAGE/TLR4 and NF-κB; elevated serum HMGB1 and reduced heart weight in tumor-bearing rats	In vitro (mechanism); animal, in vivo (serum HMGB1, heart weight)	H9c2 cardiomyoblasts exposed to ascites from RCN9 colon cancer-bearing rats; RCN9 tumor-bearing F344 rats	[46]
Hypotheses without direct evidence in patients with cancer			
Reduced cardiac output causing splanchnic hypoperfusion, impaired nutrient absorption, and accelerated cachexia progression	Hypothesis extrapolated from chronic heart failure	Not studied in patients with cancer	[50]
Reduced myocardial oxygen delivery (capillary rarefaction, anemia) contributing to the substrate shift in the cachectic heart	Hypothesis based on anemia and a non-significant decrease in capillary density (*p* = 0.07)	Orthotopic ovarian cancer mice	[28]
Cytokine release from the failing heart aggravating cancer cachexia	Hypothesis based on cardiac IL-6 in C26 mice and cytokine data in heart failure	Not determined in patients with cancer	[48,53,54]

The type of evidence refers to the design of the cited studies and does not represent a formal grading of evidence quality. ↑, increased; ↓, decreased. Abbreviations are listed at the end of the manuscript.

## 5. Proposed Hemodynamic, Neurohormonal, and Inflammatory Consequences of Cardiac Dysfunction

### 5.1. Hemodynamic Consequences: Skeletal Muscle and Splanchnic Hypoperfusion

Few human studies have examined whether cardiac dysfunction accelerates the progression of cancer cachexia. Most of the evidence discussed in this section was obtained in patients with chronic heart failure; its application to cancer cachexia is based on analogy and remains to be tested (Table 1). In cancer cachexia, loss of LV mass and impaired cardiac performance may reduce cardiac output to a degree that sufficiently compromises peripheral tissue perfusion. However, the extent to which this cardiac impairment worsens exertional fatigue and exercise intolerance cannot be determined from available data. In the Canadian study of treatment-naive patients with lung cancer described in Section 2.2, cardiac disorders and diastolic dysfunction were associated with aggravated fatigue and dyspnea, and baseline cardiac status was considered a potential source of heterogeneity in anti-cachexia trials [17].

In chronic heart failure, progressive reductions in cardiac output during exercise are accompanied by corresponding reductions in leg blood flow, and the magnitude of both reductions correlates with the severity of exercise intolerance [50]. Analogously, reduced cardiac output may impair splanchnic perfusion and intestinal function, though no direct evidence links this cardiac performance to mesenteric hypoperfusion, impaired nutrient absorption, or accelerated cachexia progression in cancer patients. The proposed heart-gut-muscle interaction in this context has not been studied prospectively in this population. 

### 5.2. Neurohormonal Changes: RAAS Activation and Sympathetic Activation

Among cachectic heart failure patients, both the renin–angiotensin–aldosterone system and the sympathetic nervous system are activated, with elevated plasma renin activity, aldosterone, norepinephrine, and epinephrine compared with healthy controls [30,31]. Since renin is rate-limiting for angiotensin II generation, this raised renin activity is compatible with the elevated plasma angiotensin II levels reported in a proportion of patients with heart failure despite ACE inhibitor therapy [51,52]. Sympathetic overactivation in cachectic patients, reflected by markedly raised plasma norepinephrine and epinephrine, contributes to a catabolic metabolic shift [30]. These data were obtained in patients with chronic heart failure; whether cardiac wasting in patients with cancer leads to comparable neurohormonal activation remains unclear. 

### 5.3. Cardiac Cytokine Release as a Secondary Inflammatory Source

The failing heart synthesizes TNF-α de novo, with circulating levels of both TNF-α and IL-6 raised in patients with heart failure [53,54]. In explanted hearts, TNF-α protein was detected in 55% of hearts from patients with dilated cardiomyopathy and in 40% of those from patients with ischemic heart disease, whereas neither TNF-α mRNA nor protein was detected in non-failing myocardium [53]. Circulating IL-6 rises with heart failure severity [54]. In multivariate Cox regression, IL-6 was an independent predictor of mortality, alongside norepinephrine and reduced left ventricular ejection fraction [54]. In skeletal muscle, TNF-α and IL-6 engage distinct intracellular cascades that culminate in cancer cachexia-associated wasting: TNF-α acts through NF-κB, whereas IL-6 signals via STAT3, p38 and FoxO downstream of glycoprotein 130 [1]. IL-6 itself is markedly upregulated in cardiac tissue of C26 tumor-bearing preclinical models [48]. Whether this cardiac inflammatory milieu aggravates cachexia progression in patients has not been determined. 

Overall, the evidence reviewed here differs in strength. In patients with advanced cancer, low LV mass has been associated with poor functional status and higher all-cause mortality [16]; previously undiagnosed cardiac abnormalities have been associated with fatigue and dyspnea [17]. These observational associations do not show that cardiac dysfunction accelerates cachexia progression. The reverse pathway, from cardiac dysfunction to skeletal muscle wasting through hypoperfusion, neurohormonal activation, or cardiac cytokine release, is extrapolated from chronic heart failure [30,31,50,51,52,53,54] (Figure 1). It therefore remains a hypothesis that requires prospective validation with serial assessment of cardiac function and body composition.

## 6. The Convergent Cardiac Burden in Chinese Cancer Patients: Cachexia, Comorbidity, and Treatment-Related Toxicity

In China, digestive system cancers, in which cachexia is especially prevalent [6], account for 41.6% of new cancer cases and 49.3% of cancer deaths [57]. The Chinese cancer population also carries a substantial burden of cardiovascular risk factors and cardiovascular disease (Section 2.1) [12,13]. In China, platinum-containing regimens are the standard first-line chemotherapy for gastric cancer [58]; for advanced hepatocellular carcinoma, the guideline-recommended regimen is FOLFOX4, a combination of oxaliplatin, fluorouracil, and leucovorin [59]. In a retrospective analysis of 932 patients receiving cisplatin-based chemotherapy, 18.1% developed a thromboembolic event during treatment or within four weeks of the last dose, with gastric and gastro-esophageal junction cancers among the higher-incidence subgroups; notably, this risk appears substantially lower with oxaliplatin-based regimens [60]. Cachexia-related cardiac change, pre-existing cardiovascular disease, and treatment-related cardiotoxicity may therefore occur together in the same patient, yet this convergent cardiac burden has not been clarified in Chinese cohorts (see Section 3 for the distinction between these conditions).

Cardiac evaluation is not routinely integrated into oncology practice in China [61,62], and, to our knowledge, prospective data on cachexia-related cardiac involvement in Chinese patients with cancer have not been reported. Cardio-oncology in China has developed mainly around the cardiotoxicity of anticancer therapy, beginning with the 2011 Chinese expert consensus on the prevention and treatment of anthracycline cardiotoxicity [62]. Cardio-oncology clinics have since been established in general, cancer, and cardiac hospitals [61], but only a few hospitals have dedicated clinics, and most of these are located in cardiac hospitals [62]. In the Canadian cohort of treatment-naive patients with lung cancer (Section 2.2), previously undiagnosed cardiac abnormalities were identified in 27.1% of patients [17]. However, the prevalence and prognostic significance of cachexia-related cardiac involvement—as distinct from already-diagnosed cardiovascular disease—in Chinese cancer patients remain unknown. The selection of patients for prospective studies is discussed in Section 7.

## 7. Clinical Implications: Cardiac Assessment in Cancer Cachexia as a Question for Prospective Research

Current multimodal management of cancer cachexia includes nutritional support, exercise, symptom control, and pharmacological treatment where indicated [20,63], with no consideration given to cardiac involvement. The diagnostic criteria for cancer cachexia are defined by weight loss, body mass index, and skeletal muscle depletion, together with reduced food intake and systemic inflammation; no consideration is given to cardiac atrophy or dysfunction [5]. Among patients with advanced cancer, low left ventricular mass has been associated with reduced stroke volume, increased resting heart rate, and impaired physical performance [16,22], as well as higher all-cause mortality [16].

Fatigue, dyspnea, and reduced exercise tolerance are common to cancer cachexia and cardiac dysfunction; these symptoms may therefore reflect cardiac dysfunction as well as skeletal muscle wasting [16,17]. Whether combined follow-up of cachexia and cardiac status improves clinical management has not been evaluated. Pending such evidence, cardiac abnormalities detected during usual clinical care are managed according to existing cardio-oncology recommendations, with referral to a cardio-oncology service or a cardiologist, where available [27], alongside the nutritional and rehabilitation support recommended for cancer cachexia [20,63]. At present, no pharmacological treatment is recommended as standard care for the underlying cachexia syndrome; however, short-term corticosteroids can be considered for appetite stimulation, while progestins, such as megestrol acetate or medroxyprogesterone acetate, can improve appetite and body weight, with important safety limitations [20,63]. Although these strategies have not yet been validated in patients, preclinical studies suggest that cachexia-related muscle wasting may be therapeutically modifiable: bisoprolol or spironolactone attenuated left ventricular mass loss, mitigated cardiac dysfunction, and improved survival in AH-130 tumor-bearing rats [25], whereas soluble ActRIIB-Fc reversed skeletal-muscle wasting together with cardiac atrophy, thereby prolonging survival in C26 tumor-bearing mice [56]. These findings suggest that skeletal and cardiac muscle wasting may represent potential therapeutic targets in cancer cachexia; however, clinical evidence validating these interventions or targets in cancer patients is still lacking. Two factors limit the translation of these findings. First, C26 tumor-bearing mice develop cachexia within approximately 17 to 19 days after tumor implantation [24,33], a far shorter time course than that observed clinically. Second, the regulation of atrophy-related genes may differ between species: human iPSC-derived cardiac muscle upregulated atrogin-1 but not MuRF-1 after cachexia induction [55], whereas C26 mouse hearts showed induction of both [23]. Despite these limitations, the authors of the AH-130 rat study have suggested clinical trials of bisoprolol and spironolactone [25]; cardiac wasting has also been proposed as an endpoint for cardio-oncology trials [22].

In China, traditional Chinese medicine is widely used among cancer patients, primarily for symptom relief, appetite improvement, and mitigation of treatment-related adverse effects. Xu et al. reviewed the available evidence on traditional Chinese medicine for cancer cachexia and reported potential benefits for appetite, body weight, inflammatory markers, Karnofsky performance status, cachexia-related symptoms, and quality of life, although the quality of evidence remains limited [64]. Whether traditional Chinese medicine affects cachexia-related cardiac wasting or cardiac dysfunction has not been studied.

**Figure 2:**
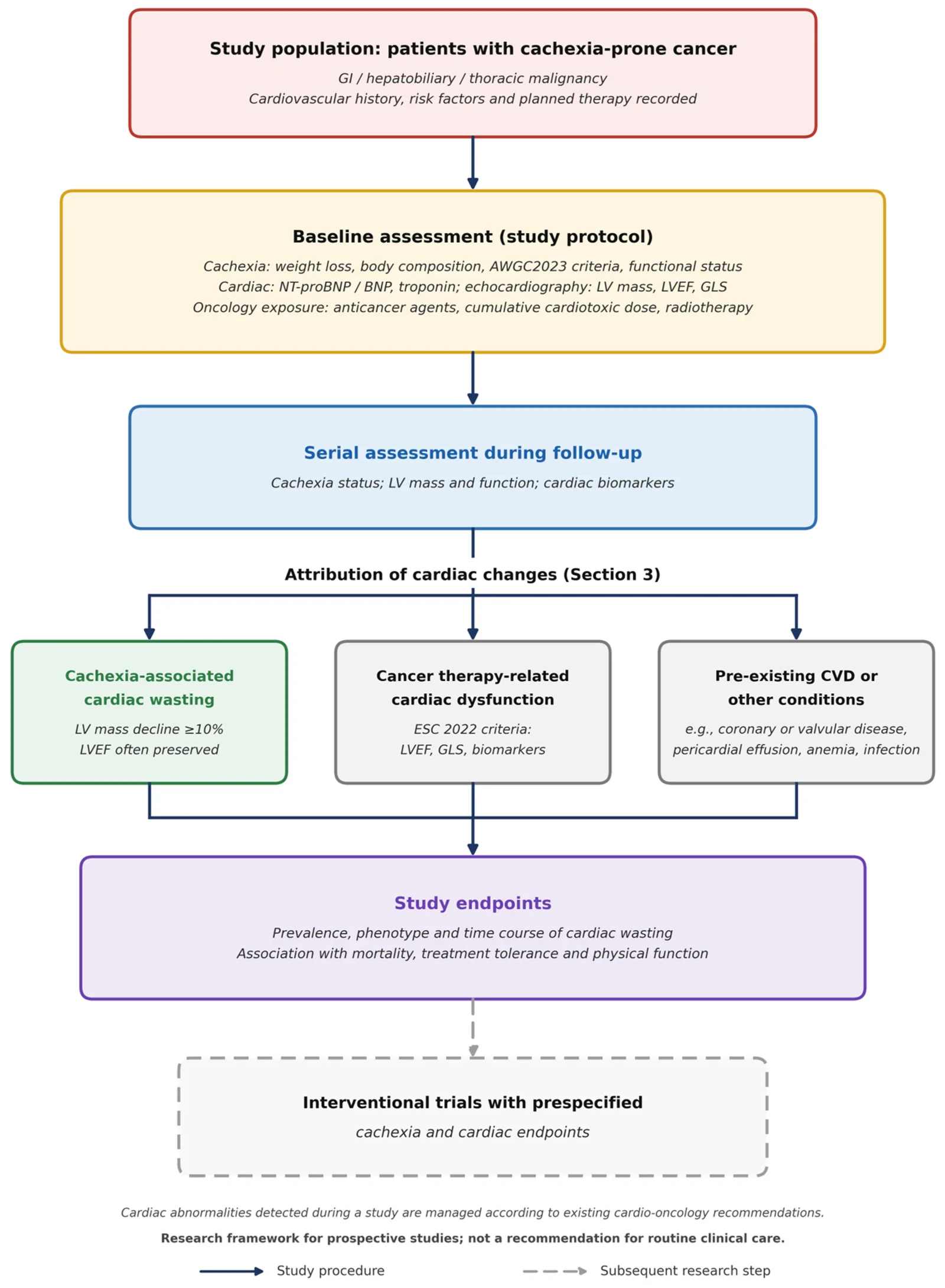
Proposed research framework for cardiac assessment in prospective studies of patients with cancer cachexia. The framework outlines the study population, baseline and serial assessments, the attribution of cardiac changes to cachexia-associated cardiac wasting, cancer therapy-related cardiac dysfunction, or pre-existing cardiovascular disease and other conditions, and the study endpoints. It is intended for prospective research and is not a recommendation for routine clinical care. Cachexia is assessed according to the AWGC2023 criteria [7,8]. The cardiac measures and the definition of cardiac wasting are based on previous echocardiographic studies [16,22]. Cancer therapy-related cardiac dysfunction is defined according to the 2022 ESC guidelines on cardio-oncology, which also guide the management of cardiac abnormalities detected during a study [27]. Other conditions to be considered include coronary or valvular disease, pericardial effusion, anemia, and infection [16,17,22,28]. Box colors distinguish the steps of the framework; in the attribution step, the green box denotes cachexia-associated cardiac wasting, whereas the grey boxes denote alternative causes of cardiac change. Solid arrows indicate study procedures; the dashed arrow and the dashed box indicate a subsequent research step. This schematic was drafted and revised during peer review with the assistance of generative AI tools (Claude Fable 5 and Claude Opus 5.5, Anthropic) and was subsequently reviewed, corrected and edited by the authors, who take full responsibility for its content; the prompts used are provided in the Supplementary Materials (Text S1).

To date, no standardized protocol exists for cardiac monitoring in cancer cachexia. Whether earlier cardiac assessment can guide treatment selection, enable timely cardioprotective intervention, or improve patients’ outcomes should be evaluated in further prospective studies. Until such data are available, routine cardiac assessment of patients with cancer cachexia cannot be recommended outside clinical studies; the approach outlined in Figure 2 is therefore presented as a framework for prospective research.

The elements of Figure 2 are based on the data reviewed above. Patients with gastrointestinal, hepatobiliary, or thoracic tumors are proposed as the study population: pancreatic, gastric, and esophageal cancers show the highest prevalence of cachexia in Chinese cohorts [6,7,14], lung cancer is a leading cause of cancer death in China [4], and, apart from lung cancer, these tumor types have not been examined specifically in studies of cardiac involvement [16,17]. Cardiovascular history and exposure to cardiotoxic treatment are recorded at baseline so that cardiac wasting can be distinguished from other causes of cardiac dysfunction (Section 3). Cardiovascular comorbidity is common among Chinese patients with cancer [12,13] and was an exclusion criterion in the studies that defined cardiac wasting [16,22]; platinum-based chemotherapy, a standard treatment for gastric cancer and advanced hepatocellular carcinoma in China [58,59], is associated with cardiovascular toxicity [60,65]. Echocardiographic LV mass is the principal cardiac measure, as the operational definition of cardiac wasting is based on it [16,22]; LVEF, GLS, cardiac troponin, and natriuretic peptides are added to identify CTRCD according to the ESC definition [27]. The framework has not been tested; its assessments and follow-up intervals will need to be adapted to the setting of each study.

## 8. A Cardiac Cachexia Research Agenda for China

In experimental models of cancer cachexia, cardiac atrophy and skeletal muscle atrophy share several catabolic mechanisms, including activation of the ubiquitin–proteasome system and autophagy, in the setting of systemic inflammation and tumor- or immune-derived mediators [1]. Cardiac wasting has also been associated with neurohormonal activation, including sympathetic and renin–angiotensin–aldosterone system signaling: plasma noradrenaline, renin, and aldosterone were increased in tumor-bearing rats, and plasma renin and aldosterone were increased in patients with cancer [2,25]. Yet Chinese studies on cancer cachexia have mainly centered on prevalence, diagnosis, nutritional status, and prognosis, with limited attention to cardiac changes and underlying mechanisms [6,7,14] (Table 2). INSCOC, the largest Chinese oncology nutrition cohort used for cancer-cachexia analyses, was designed to characterize nutritional status and its clinical outcomes across multiple cancer types, but cardiac structure, cardiac function, and cardiac biomarkers were not included as study endpoints [7,66]. Cardiac involvement in Chinese patients with cancer cachexia therefore remains unclear at both the structural and the functional level, and its contribution to mortality or chemotherapy tolerability has not been quantified. 

Addressing this gap requires prospective studies with longer-term serial assessment of cardiac structure and function. Most mechanistic evidence discussed in Section 4 and Section 5 comes from rodent models of cancer cachexia, particularly C26-based models; whether the same mechanisms operate in human cardiac wasting is not yet clear. A recent preprint using human iPSC-derived cardiac muscle reported upregulation of atrogin-1, but not MuRF-1, after cachexia induction, diverging from C26 mouse studies in which both E3 ligases are induced [55]. To date, the detailed prospective echocardiographic data on cardiac wasting available come mainly from Charité cohorts, in which follow-up echocardiography was performed in subsets of patients after approximately four to five months [16,22]. These studies provide important longitudinal evidence, but follow-up was necessarily restricted to patients who survived and were able to undergo repeat assessment. The key human studies cited in this review, including their limitations, are summarized in Table 3. Moreover, gastric, esophageal, and hepatocellular cancers, which carry a substantial burden of cachexia and cancer mortality in China, have not been specifically examined in clinical studies of cardiac involvement: one study was restricted to non-small cell lung cancer, and the other did not recruit any patients with hepatocellular cancer [16,17]. Future studies in these populations should integrate serial echocardiography and cardiac biomarkers with standard cachexia assessment to define the prevalence, temporal progression, and prognostic significance of cardiac involvement, and to evaluate its association with mortality and anticancer treatment tolerance.

Population-specific factors also need to be considered. The AWGC2023 criteria use a weight-loss threshold of 2% and a BMI cut-off of 21 kg/m^2^, reflecting the lower average BMI of Asian populations and the different contribution of BMI to chronic disease compared with Caucasian populations [8]. Within China, the prevalence of cachexia was 36.3% in Han patients and 38.3% in patients of ethnic minorities, with significant differences in nine of 16 cancer types [6]. To our knowledge, no study has compared cardiac wasting, or the myocardial expression of atrophy-related genes such as MuRF-1 and atrogin-1, between patients of different ancestry; such comparisons could be included in future prospective cohorts.

Once the prevalence, at-risk subgroups, and temporal relationship between cachexia and cardiac involvement are established through observational studies, interventional trials should incorporate body weight, skeletal muscle mass, and functional status alongside prespecified cardiac endpoints, including left ventricular mass, systolic function, and cardiac biomarkers. Regulatory and clinical-trial reviews in cachexia have emphasized that such trials require both body-composition and functional endpoints, since changes in body weight or muscle mass alone do not reliably reflect clinical benefit [67]. These trials should determine whether cachexia-directed therapy prevents, attenuates, or reverses LV mass loss or preserves cardiac function, and whether interventions targeting cardiac wasting improve body composition, physical performance, quality of life, or survival in patients with cancer cachexia.

Across study designs, cancer-associated cardiac wasting should be distinguished from treatment-related cardiotoxicity, pre-existing cardiovascular disease, and non-cardiac systemic conditions (Section 3), as these conditions may coexist in the same patient but differ in etiology, structural pattern, and timing relative to cancer diagnosis and treatment. Without careful phenotyping, cardiac changes caused by anticancer therapy or pre-existing cardiovascular disease could otherwise be interpreted as cachexia-related cardiac wasting. Lena et al. [16] showed that low LV mass was not explained by previous cardiotoxic anticancer therapy, whereas Anker et al. [22] reported similar anticancer therapy-naïve status across LV mass quartiles but did not specifically isolate the effects of treatment type, cumulative exposure, or treatment duration. The latter study also acknowledged that cancer type as well as the strategy and duration of anticancer therapy may influence the progression of cachexia and cardiac wasting. Platinum-based regimens are widely used across several malignancies and are associated with potential cardiovascular toxicity, including myocardial ischemia, arrhythmias, hypertension, and thromboembolic events [65]. Among platinum agents, cisplatin-based chemotherapy has been associated with a high incidence of thromboembolic events (Section 6) [60]. Future studies should therefore record specific anticancer agents, cumulative doses of potentially cardiotoxic drugs, and treatment duration alongside serial cardiac assessment, so that cachexia-related cardiac changes can be distinguished from pre-existing cardiovascular disease and treatment-related cardiotoxicity.

## 9. Conclusions

In China, cancer cachexia has so far been studied mainly in relation to prevalence, diagnostic criteria, nutritional status, functional impairment, medical burden, and prognostic significance, while cardiac involvement has rarely been addressed in Chinese cancer-cachexia cohorts [6,7]. Whether structural or functional myocardial abnormalities occur in Chinese patients with cancer cachexia, and whether they are associated with adverse clinical outcomes, therefore, has not been clarified. Addressing this gap in Chinese patients is warranted for four reasons, which relate to the size of the population at risk and to the conditions for clinical study rather than to evidence of a distinct cardiac phenotype: digestive system cancers, which include tumor types with a high prevalence of cachexia, account for a large proportion of new cancer diagnoses in China [6,7,57]; cardiovascular risk factors and disease are common among Chinese cancer patients [12]; cardiotoxic anticancer treatments may complicate the interpretation of cachexia-related cardiac changes [27]; and cardio-oncology services in China, although developing, are not yet widely available across the whole country [61,62].

In non-Chinese cohorts of patients with advanced cancer, low left ventricular mass has been associated with lower stroke volume and cardiac output, although left ventricular ejection fraction was frequently preserved [16,22]. Left ventricular mass was lowest in patients with cachexia and independently predicted poor survival [16], suggesting that cardiac wasting may be of prognostic relevance. Preclinical studies, mainly in rodent models of cancer cachexia, have proposed several mechanisms underlying cardiac wasting, including ubiquitin–proteasome–mediated proteolytic activation (↑MuRF-1, ↑atrogin-1, ↑protein ubiquitination) in the colon-26 model [23]; suppressed mTOR-dependent anabolic signaling (↓p-mTOR, ↓p-S6, ↓p-4EBP1) with concurrent AMPKα activation, despite elevated Akt phosphorylation, in the ApcMin/+ model [40]; metabolic and mitochondrial remodeling (a redox shift toward glycolysis with ↑HIF-1α, impaired PINK1/Parkin-mediated mitophagy, and reduced glutathione-peroxidase content) in the Lewis lung carcinoma (LLC) model [49]. However, these mechanisms have not been confirmed in human disease. A recent preprint using human iPSC-derived cardiac muscle reported upregulation of atrogin-1 but not MuRF-1 across both C26- and HCT116-induced cachexia models [55], differing from C26 mouse studies in which both E3 ligases were transcriptionally upregulated (with MuRF-1 also elevated at the protein level) [23]. As cardiac involvement in cancer cachexia has, to our knowledge, not been examined in Chinese cohorts, neither the wasting phenotype nor its related molecular pathways can be assumed to apply directly to Chinese patients. Future studies should therefore combine patient-derived or iPSC-derived cardiomyocyte models with paired blood samples and serial cardiac assessment to determine whether mechanisms identified in rodent models are present in Chinese patients and whether they lead to similar or distinct downstream changes.

In parallel, to address the absence of data on cardiac involvement in Chinese oncology patients, prospective longitudinal cohort studies should be conducted in patients with cachexia-prone malignancies, particularly gastrointestinal, hepatobiliary, and thoracic tumors, in which serial cardiac evaluation is included in the study protocol together with standard cachexia assessment: serial measurements of left ventricular mass, stroke volume, cardiac output, systolic and diastolic function, as well as cardiac biomarkers. These cardiac measures should be interpreted alongside weight loss, body composition, inflammatory status, and functional status, together with a complete record of cardiovascular history and exposure to cardiotoxic treatment, so that cachexia-associated cardiac wasting can be distinguished from pre-existing cardiovascular disease and treatment-related cardiotoxicity (Table 4). Once prevalence, phenotype, and timing are established, interventional trials with prespecified cachexia and cardiac endpoints could help determine whether cachexia-directed or cardioprotective treatment preserves left ventricular mass and its function, and whether doing so improves treatment tolerance, physical performance, or survival. This sequence of observational and interventional studies is needed to determine whether early detection of cardiac wasting or dysfunction provides a basis for refining treatment strategy and improving outcomes in Chinese patients with cancer cachexia.

## Figures and Tables

**Table 2:** Evidence gaps in Chinese cancer cachexia research.

Domain	Existing Evidence in China	Cardiac Endpoints Included?	Main Gap
Prevalence	National and advanced-cancer cohorts report cachexia in more than one third of patients [6,7,14].	No	No Chinese estimate of cardiac wasting prevalence.
Diagnostic criteria	AWGC2023 criteria [8] predict survival and medical burden in Chinese patients [7].	No	No integration of LV mass, cardiac biomarkers, or cardiac function.
Machine learning	Models can identify cachexia when weight-loss history is unavailable [9,15].	No	Cardiac variables have not been included as predictors or outcomes.
Cardio-oncology	Practice and guidelines focus mainly on treatment-related cardiovascular toxicity [61,62].	Partly	Cardiac wasting has not been evaluated as a surveillance target in prospective studies.
Clinical outcomes	Cachexia and cardiovascular comorbidity separately predict poor outcomes [7,12].	No	Independent contribution of cardiac wasting to mortality, treatment tolerance, and function not studied in Chinese patients.

**Table 3:** Key human studies cited in this review: design, findings, and limitations.

Study	Design and Setting	Population	Main Findings	Limitations
Cachexia prevalence in China				
Sun et al., 2015 [14]	Cross-sectional survey; single centre, Wuhan, China; October–December 2012	390 patients with advanced cancer	Cachexia in 35.9%; pancreatic 88.9%, gastric 76.5%, esophageal 52.9%; 76.4% of cases not recognized by treating physicians	Single centre; hospitalized patients only; recruitment over 3 months; no cardiac assessment
Li et al., 2022 [6]	Multicentre prospective cohort (INSCOC); 80 tertiary hospitals in China; 2012–2020	47,604 patients with 16 common cancers	Cachexia in 37.0% (2011 international consensus); highest in pancreatic cancer (63.3%), lowest in breast cancer (15.4%)	Tertiary hospitals only; observational design; no cardiac assessment
Xie et al., 2024 [7]	Retrospective post hoc analysis of INSCOC	5426 patients with cancer	Cachexia in 43.8% (AWGC2023 criteria); pancreatic 68.9%, gastric 57.7%, esophageal 54.0%; cachexia independently associated with survival and with prolonged hospitalization and high medical expenses	Chinese patients only; no external validation cohort; no cardiac assessment
Cardiovascular comorbidity in Chinese patients with cancer				
Liu et al., 2019 [12]	Multicentre hospital-based cohort; four cancer specialty hospitals in China; 1995–2018	710,170 patients with cancer	Cardiovascular risk factors in 13.0% and CVD in 5.0%; most conditions associated with higher all-cause mortality, dyslipidemia with lower mortality	Time of onset of comorbidity not recorded; multivariable analysis restricted to 62,549 patients with complete data; causes of death not available
Li et al., 2021 [13]	Cross-sectional; single centre, Dalian, China; 2008–2018	35,861 patients with lung, colorectal, gastric, breast, or thyroid cancer	Hypertension in 21.9%, coronary heart disease in 6.5%	Single centre; five cancer types; cachexia not assessed
Cardiac involvement in patients with cancer				
Kazemi-Bajestani et al., 2019 [17]	Cross-sectional; Edmonton, Canada; 2013–2016	70 treatment-naive patients with metastatic NSCLC	Clinically relevant cardiac disorders in 12.9% and diastolic dysfunction with preserved LVEF in 14.3%; associated with fatigue and dyspnea	Small sample; single tumor type; reduced LVEF likely due to pre-existing ischemic heart disease in seven patients
Lena et al., 2023 [16]	Prospective cohort; single centre, Berlin, Germany; 2017–2020	300 patients with mostly advanced cancer; 60 healthy controls; 60 patients with heart failure	LV mass lower than in controls and lowest in cachexia; independent predictor of all-cause mortality; further decline of 9.3% ± 1.4% after approximately 4 months (*n* = 90)	Single centre; significant CVD, LVEF < 50%, and infection excluded; follow-up echocardiography only in a subset of survivors
Anker et al., 2025 [22]	Prospective cohort; same centre as [16]; 2017–2023	398 patients with active, mostly advanced cancer	Low LV mass associated with smaller chamber volumes, thinner walls, and lower stroke volume and cardiac output; LVEF and GLS similar across LV mass quartiles	Recruitment overlapping with [16]; same exclusion criteria; follow-up in a subset (*n* = 143)

CVD, cardiovascular disease; GLS, global longitudinal strain; INSCOC, Investigation on Nutrition Status and Clinical Outcome of Common Cancers; LV, left ventricular; LVEF, left ventricular ejection fraction; NSCLC, non-small cell lung cancer.

**Table 4:** Proposed minimum dataset for prospective Chinese cardiac-cachexia cohorts.

Category	Suggested Measures	Purpose
Cachexia phenotype	Weight trajectory, dietary intake, appetite symptoms, body composition, handgrip strength, ECOG/performance status	Define cachexia severity and functional impact.
Inflammation and nutrition	CRP, albumin, hemoglobin, neutrophil-to-lymphocyte ratio, inflammatory burden index where available	Link systemic inflammation and nutritional reserve to cardiac changes.
Cardiac biomarkers	NT-proBNP/BNP, troponin when clinically indicated, resting heart rate, blood pressure	Screen for myocardial stress or injury and hemodynamic adaptation.
Echocardiography	LV mass, LV mass indexed to height, LV volumes, wall thickness, stroke volume, cardiac output, LVEF, diastolic function, GLS	Characterize cardiac wasting beyond LVEF.
Oncology exposure	Cancer type/stage, tumor burden, systemic therapy, radiotherapy, cumulative cardiotoxic dose, treatment timing	Separate cachexia-associated cardiac wasting from treatment-related toxicity.
Clinical outcomes	Treatment interruption, hospitalization, functional decline, quality of life, survival	Test whether cardiac involvement is clinically actionable.

## Data Availability

No new data were created or analyzed in this study. Data sharing is not applicable to this article.

## Supplementary Materials

The following supporting information can be downloaded at https://www.mdpi.com/article/10.3390/curroncol33100635/s1. Text S1: Prompts used to generate Figure 1 and Figure 2 with generative AI tools.

## Abbreviations

The following abbreviations are used in this manuscript:

Ang II	angiotensin II
atrogin-1	muscle atrophy F-box protein
AWGC2023	Asian Working Group for Cachexia 2023 consensus
Beclin-1	BCL2-interacting protein 1
CTRCD	cancer therapy-related cardiac dysfunction
CVD	cardiovascular disease
ESC	European Society of Cardiology
FoxO3	forkhead box protein O3
GI	gastrointestinal
GLS	global longitudinal strain
HMGB1	high-mobility group box 1 protein
IL	interleukin
iPSC	induced pluripotent stem cell
LC3	microtubule-associated protein light chain 3
LV	left ventricular
LVEF	left ventricular ejection fraction
MHC	myosin heavy chain
mTOR	mechanistic target of rapamycin
MuRF-1	muscle RING-finger protein-1
NF-κB	nuclear factor-κB
NT-proBNP	N-terminal pro-B-type natriuretic peptide
RAAS	renin–angiotensin–aldosterone system
SNS	sympathetic nervous system
TNF-α	tumor necrosis factor-α
UPS	ubiquitin–proteasome system
