# Supplementary material for: Cancer Cachexia and the Heart: A Review with Focus on China
Source: Curr Oncol. 2026 Oct 20;33(10):635. doi: 10.3390/curroncol33100635 (PMC13702992; doi:10.3390/curroncol33100635)
Supplement: Supplementary file 1 [file curroncol-33-00635-s001.zip › curroncol-4502193-supplementary.pdf]

Supplementary Materials

Cancer Cachexia and the Heart: A Review with Focus on China

Siqi Wang, Zihao Chen, and Jochen Springer

Manuscript ID: curroncol-4502193

Text S1. Prompts used to generate Figures 1 and 2 with generative AI tools.

Draft versions of the two schematic figures were generated with Claude Fable 5 (Anthropic; API model identifier claude-fable-5). During peer review, Figure 2 was redrawn with Claude Opus 5.5 (Anthropic; API model identifier claude-opus-5-5) to convert the proposed clinical pathway into a framework for prospective research. The authors reviewed, corrected, and edited all outputs and take full responsibility for the content of the figures. The prompts are reproduced below as used.

Figure 1 (Claude Fable 5)

Create a clean, publication-quality mechanism flowchart (white background, rounded rectangular boxes with colored borders and pale fills, navy solid arrows and grey dashed arrows) titled conceptually "Mechanisms underlying cardiac wasting and dysfunction in cancer cachexia". Top box (red border): "Tumor- and immune-derived signals" — TNF-α, IL-6 (NF-κB), IL-1; myostatin / activin A; HMGB1, cachexokines (e.g. Ataxin-10). This top box points with solid arrows down to two green boxes: "Skeletal muscle wasting" (UPS · autophagy · atrophy) on the left, and "Cardiac wasting" (UPS · autophagy · atrophy) on the right. "Cardiac wasting" points down to a purple box "Molecular mechanisms (heart)" listing: ↑ MuRF-1 / atrogin-1, ↑ ubiquitination (UPS); ↑ Beclin-1 / LC3 autophagy (FoxO3); ↓ mTOR-dependent protein synthesis; Fetal gene re-expression (MHCα→MHCβ); Metabolic & mitochondrial remodeling; Contractile & calcium-handling deficits; HMGB1-driven apoptosis / autophagy; and in grey "Human iPSC: atrogin-1↑ but not MuRF-1 [65]". A separate grey box on the left, "Cardiotoxic therapy" (Platinum chemotherapy, radiotherapy; "(separate, established cause)" in grey), points via solid arrow toward the central red box. Both the purple "Molecular mechanisms" box and the grey "Cardiotoxic therapy" box converge with solid arrows into a central red box "Cardiac dysfunction" (↓ LV mass, ↓ stroke volume, ↓ cardiac output; LVEF / GLS often preserved). "Cardiac dysfunction" fans out with three solid arrows to three blue boxes: "Hypoperfusion" (↓ skeletal muscle & splanchnic (gut) flow); "Cardiac cytokine release" (failing heart → TNF-α, IL-6; "(effect on cachexia undetermined)" in grey); "Neurohormonal activation" (RAAS / SNS: ↑ Ang II, catecholamines). These three blue boxes point via grey dashed arrows down to an orange box "Aggravated cachexia progression" (↑ skeletal muscle wasting, ↑ mortality). A long grey dashed "Feedback loop" arrow runs up the left side from "Aggravated cachexia progression" back to "Skeletal muscle wasting". Bottom legend: solid navy arrow = "Clinical or experimental support"; grey dashed arrow = "Proposed / not yet tested".

Figure 2, original version (Claude Fable 5)

Create a clean, publication-quality clinical-pathway flowchart (white background, rounded colored boxes, navy solid arrows for established steps and a grey dashed arrow for the research step) titled conceptually "Proposed clinical pathway for selective cardiac assessment in patients with cancer cachexia". Top box (red border): "Cancer patient with high cachexia risk" — GI / hepatobiliary / thoracic malignancy; pre-existing CVD or risk factors; planned cardiotoxic treatment. Arrow down to a yellow box "Baseline risk stratification" (Assess cachexia severity, cardiovascular history, and planned treatment). This box branches into two, labelled "Yes (high risk)" (left) and "No" (right): – Left, green box "Consider cardiac assessment": NT-proBNP / BNP, troponin; Echocardiography: LV mass, LVEF, GLS* [16,22]. – Right, grey box "Cachexia assessment only": Weight loss, body composition; AWGC2023 criteria; functional status [7,8]. Both branches converge with solid arrows into a blue box "Serial monitoring during treatment" (Cachexia status (AWGC2023 criteria); LV mass and function if baseline assessed [16,22]). Arrow down to

a magenta box “Fatigue or dyspnea disproportionate to weight loss” (Echocardiography ± cardiac biomarkers if not previously performed [16,17]). Arrow down to a purple box “Cardiac wasting and cachexia coexist” (Tumor-directed therapy + nutritional and rehabilitation support; Cardio-oncology or cardiology referral where available [20,60,61]; Incorporate cardiac endpoints in prospective studies). A grey dashed arrow leads down to a dashed-border box “Prospective study with serial cardiac and cachexia endpoints”. Bottom legend: solid navy arrow = “Established pathway”; grey dashed arrow = “Proposed / research pathway”.

Figure 2, version revised during peer review (Claude Opus 5.5)

The prompt used was the reviewer’s comments together with the instruction “Please revise the manuscript precisely according to each of the peer reviewer’s comments” (translated from Chinese).
